# Deaths related to post-COVID in Italy: a national study based on death certificates

**DOI:** 10.3389/fmed.2024.1401602

**Published:** 2024-07-03

**Authors:** Francesco Grippo, Giada Minelli, Roberta Crialesi, Stefano Marchetti, Flavia Pricci, Graziano Onder

**Affiliations:** ^1^National Institute of Statistics, Integrated System for Health, Social Assistance and Welfare, Rome, Italy; ^2^Statistical Service, Istituto Superiore di Sanità, Rome, Italy; ^3^Department of Cardiovascular, Endocrine-Metabolic Diseases and Aging, Istituto Superiore di Sanità, Rome, Italy; ^4^Fondazione Policlinico Gemelli IRCCS, Rome, Italy; ^5^Department of Geriatric and Orthopedic Sciences, Università Cattolica del Sacro Cuore, Rome, Italy

**Keywords:** long-COVID, post-COVID, mortality, death certificates, morbidity

## Abstract

**Introduction:**

SARS-CoV-2 infection has been associated with the onset or persistence of symptoms in the long-term after the acute infection is resolved. This condition known as Post-COVID, might be particularly severe and potentially life-threatening. However, little is known on the impact of post-COVID condition on mortality. Aim of the present study is to assess and quantify Post-COVID deaths in Italy in years 2020 and 2021, based on an analysis of death certificates.

**Methods:**

Data from the Italian National Cause of Death Register were analyzed. ICD-10 code U09.9, released by the World Health Organization in September 2020, was used to identify the ‘Post-COVID’ condition. Numbers of post-COVID deaths from October 2020 to December 2021 were analyzed. Rates of post-COVID deaths were calculated for the year 2021.

**Results:**

Between October 2020 and December 2021, 4,752 death certificates reporting post-COVID condition were identified. Of these, 14.9% (*n* = 706) occurred between October and December 2020 and 85.1% (*n* = 4,046) in 2021. In 46.0% of post-COVID-related deaths, the underlying cause of death was COVID-19. Other frequent underlying causes were heart disease (14.3% of cases), neoplasms (9.2%), cerebrovascular diseases (6.3%) and Alzheimer’s disease and other dementias (5.5%). The mortality rate related to post-COVID conditions in year 2021 was 5.1 deaths per 100 thousand inhabitants and it increased with increasing age. Men showed a higher mortality rate than women (4.3 deaths per 100 thousand in women and 6.0 deaths per 100 thousand in men).

**Discussion:**

Post-COVID conditions contributed to a substantial number of deaths in Italy. Strategies to identify the population at risk of severe long-term consequences of SARS-CoV-2 infection and interventions aimed at reducing this risk must be developed.

## Introduction

Severe acute respiratory syndrome coronavirus 2 (SARS-CoV-2) infection has caused numerous deaths worldwide. International data show that since the beginning of SARS-CoV-2 epidemic more than 7 million persons died because of Coronavirus disease (COVID-19) worldwide (1). In particular, SARS-CoV-2 infection can cause an acute respiratory syndrome associated with high mortality rate particularly in old and frail persons and in those with a high multimorbidity burden (2, 3).

SARS-CoV-2 infection has also been associated with the onset or persistence of symptoms in the long-term, weeks or months after the acute infection is resolved. This condition known as Post-COVID is defined as the persistence of symptoms due to SARS-CoV-2 infection for more than 12 weeks after the start of acute symptoms (4, 5). The post-COVID condition was officially recognized by the World Health Organization (WHO) in 2020 by the definition in the International Classification of Diseases, 10th Revision (ICD–10) of mortality code U09.9 for coding and reporting Post-COVID conditions linked with preceding acute COVID-19 (6). According to a meta-analysis of 194 studies including 735,006 individuals, 45% of SARS-CoV-2 infection survivors experienced at least one unresolved condition at a mean follow-up of 4 months (7). Post-COVID has also been associated with poor quality of life and with a high utilization healthcare services, including outpatient visits, diagnostic tests and hospitalizations (8, 9). Female sex, older age, pre-existing comorbidities were found to be significantly associated with its development (10).

Although some Post-COVID conditions affecting the neurological, cardiocirculatory, respiratory and endocrine system might be particularly severe and potentially life-threatening (11, 12), little is known on the impact of Post-COVID condition on mortality. A study, performed by the Center for Disease Control (CDC) in the United States (US) and based on death certificates analysis, showed that post-COVID played a part in 3,544 deaths in the US from January 2020 through the end of June 2022 (13). However, this study was based on literal text search of death certificates, due to the fact that the code U09.9 was not implemented in the United States in the period considered in the analysis, leading potentially to an underestimation of Post-COVID deaths. Aim of the present study is to assess and quantify Post-COVID deaths in Italy in years 2020 and 2021 (those associated with the strongest impact of COVID-19 on mortality in Italy) (14), based on an analysis of codes reported in death certificates.

## Methods

### Data source

Analyses were performed based on the Italian National Cause of Death Register (15), managed by the Italian National Institute of Statistics (ISTAT), which collects information on the cause of death and demo-social variables (sex, age, residence, citizenship, etc.) for all deaths occurring in Italy. Causes of death are provided by physicians who report the sequence of causes directly leading to death and other relevant morbid conditions that may have contributed to death. All conditions reported are classified according to the International Classification of Diseases, 10th Revision (ICD-10) of the World Health Organization (WHO) (16). From the coded information, the underlying cause (UC) of death, defined as the disease or injury that initiated the sequence of morbid events leading directly to death, and other relevant conditions contributing to death are extracted. The coding and the selection of the UC is performed by means of the worldwide used software Iris.^1^ Certificates reporting COVID-19 have been coded according to the instructions issued by WHO which are incorporated also in the software Iris (6, 17). The available national data concerns deaths occurring in Italy until December 2021.

### Post-COVID and COVID-19 definitions

Since the beginning of the pandemic, the WHO has progressively activated the emergency ICD-10 codes for COVID-19 related conditions (see Table A1 in the Appendix). In addition to the mortality codes for COVID-19, the code U09.9, indicating late effects or prolonged course, was introduced in September 2020, using the neutral wording ‘Post-COVID’ (6, 18). WHO specified that ‘This term does not pre-empt any etiopathological links, and leaves space for linking any condition to a preceding acute COVID’ (6). The code U09.9 is not used for the UC and if the code U09.9 was reported as UC, according WHO provision, the death was attributed to COVID-19.

Numbers of Post-COVID deaths presented in this study refer to deaths occurring from October 2020 (after the code U09.9 for Post-COVID condition was made available by WHO) till December 2021, for which the code U09.9 is reported anywhere on the death certificate. Rates of Post-COVID deaths refer only to deaths occurring in year 2021. Confirmed cases of COVID-19 presented in the manuscript were obtained from the National Institute of Health (19).

### Data analyses

The distribution of Post-COVID deaths by UC is presented according to ICD-10 codes. Age-standardized mortality rates were computed by the direct method, using five-year age-group specific rates, except for the 0, 1–4 age groups and the upper age group 95 years and more. Age-specific rates were calculated using mid-year population in 2021. The European standard population was used for weighing the rates (20). We calculated age-standardized mortality rates for Italy as a whole and by sex and age groups (i.e., <50, 50–64, 65–79, and ≥ 80 years).

## Results

Between 1st October 2020 and 31st December 2021, 4,752 death certificates reporting Post-COVID condition were identified in the Italian National Cause of Death Register, 0.43% of the over 1.1 million deaths observed in the same period (Table A2 in the Appendix). Of these, 14.9% (*n* = 706) occurred between October and December 2020 and 85.1% (*n* = 4,046) in 2021. Overall, 46.0% of deaths were observed in men and 54.0% in women. Concerning the age distribution, 65.0% of deaths were aged 80 years or more, 26.4% 65–79 years, 7.0% 50–64 years and 1.5% were 0–49 years.

Table 1 reports the distribution of cases according to the UC of death. In 46.0% of Post-COVID related deaths, the UC was COVID-19. Other frequent UC were heart disease (14.3% of cases), neoplasms (9.2%), cerebrovascular diseases (6.3%), Alzheimer disease and other dementias (5.5%) and diseases of digestive system (2.6%). This distribution was slightly different between men and women: in men, COVID-19 and neoplasms were more commonly observed as an UC of death, while women showed a higher proportion of heart disease, cerebrovascular disease and Alzheimer and other dementias. The proportion of deaths reporting COVID-19 as UC varied by age: from 53.4% in 0–49 years old group, to 48.1% in 50–64 years, 48.6% in 65–79 years and 44.6% in 80 years and more groups.

**Table 1:** Underlying causes of post-COVID deaths in Italy in 2020 and 2021.

Underlying cause of death		Number			Percent		
ICD10 codes	Description	Men	Women	Total	Men	Women	Total
U071, U072	COVID-19	1,075	1,112	2,187	49.2	43.3	46.0
I10-I25, I30-I51	Heart diseases	272	408	680	12.4	15.9	14.3
	of which:						
I20-I25	Ischemic heart disease	135	117	252	6.2	4.6	5.3
I10-I15	Hypertensive heart diseases	49	137	186	2.2	5.3	3.9
I30-I51	Other heart diseases	88	154	242	4.0	6.0	5.1
C00-D48	Neoplasms	244	191	435	11.2	7.4	9.2
I60-I69	Cerebrovascular diseases	121	179	300	5.5	7.0	6.3
G30, F01-F03	Alzheimer disease and other dementias	75	187	262	3.4	7.3	5.5
K00-K99	Diseases of the digestive system	63	62	125	2.9	2.4	2.6
N00-N99	Diseases of the genitourinary system	42	42	84	1.9	1.6	1.8
E10-E14	Diabetes	48	50	98	2.2	1.9	2.1
V00-X59	Accidental deaths	21	53	74	1.0	2.1	1.6
A00-B99	Infectious and parasitic diseases	26	40	66	1.2	1.6	1.4
G20-G21	Parkinson disease	38	28	66	1.7	1.1	1.4
	Other causes	160	215	375	7.3	8.4	7.9
	Total	2,185	2,567	4,752	100.0	100.0	100.0

**Figure 1:**
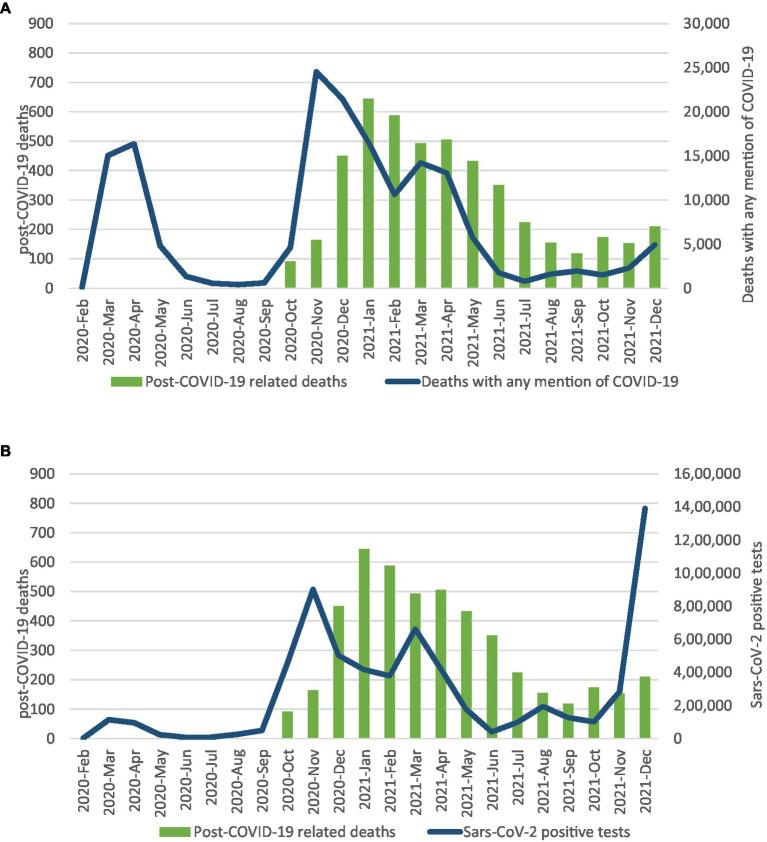
Post-COVID-19 deaths and SARS-CoV-2 positive tests by month **(A)** and post-COVID-19 and COVID-19 deaths by month **(B)**.

Figure 1B shows the distribution of Post-COVID deaths by month from Febrary 2020 to December 2021 in comparison with the monthly numbers of Sars-CoV-2 positive tests. The monthly trend of Post-COVID related deaths followed the same pattern of the number of cases with a slightly lag time for each wave of the pandemic. Post-COVID related deaths showed a peak (644 cases, the maximum monthly number) in January 2021 following the November 2020 peak of positive tests (two months lag time). A second peak of deaths occurred in April 2021 (506 cases) following the March 2021 peak of positive tests, and the third in October 2021 (173 cases) after the August peak of positive tests. A similar pattern was observed when Post-COVID related deaths were compared with COVID-19 deaths (Figure 1A).

**Figure 2:**
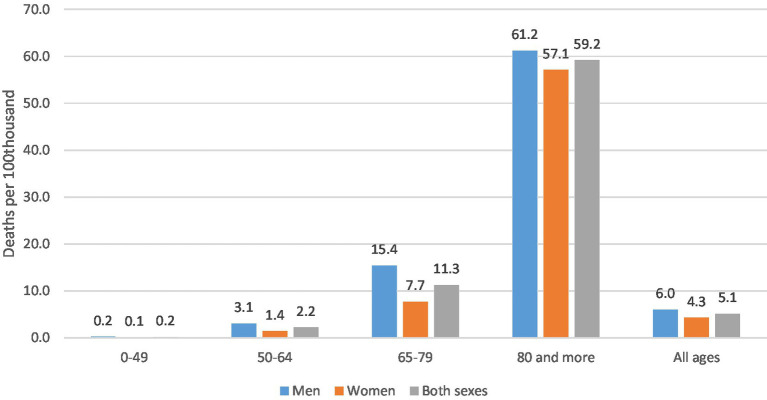
Standardized mortality rates of post-COVID condition by sex and age group. Year 2021 (deaths per 100 thousand inhabitants).

In 2021, the standardized mortality rate related to Post-COVID condition was 5.1 deaths per 100 thousand inhabitants (Figure 2) while the mortality rate for all causes was 898.5 (Table A2 in the Appendix). Based on mortality rates, Post-COVID condition accounts for 0.6% of total mortality. Mortality rate of Post-COVID condition progressively increased with increasing age, reaching 59.2 deaths per 100 thousand inhabitants in the population aged 80 years or older. In addition, men showed 40% higher value of mortality rates than women (in 2021 the rate was 4.3 deaths per 100 thousand in women and 6.0 deaths per 100 thousand in men). Gender differences were higher in younger age groups and tended to disappear only at older ages: the rate ratio male/female was 2.3 in 0–49 years old group, 2.2 in the 50–64 years group, 2.0 in the 65–79 years group and 1.1 in over-80 years group.

Post-COVID mortality rates vary across geographical areas within the Country (Table A2 in the Appendix), with decreasing gradient from North to south. Mortality rates range from 3.0 deaths per 100 thousand in the Island area (0.3% of overall mortality rate) and 6.8 (0.8% of overall mortality rate).

## Discussion

This is one of the first national studies quantifying, in terms of mortality, the burden of Post-COVID condition. Italy, in particular, faced significant challenges during the early stages of the pandemic, reporting approximately 6 million cases and over 140 thousand deaths from February 2020 (1), but the impact of long-term consequences of COVID-19 on mortality where not estimated so far. The study shows that in 2020 and 2021, Post-COVID contributed to more than 4,700 deaths in Italy. COVID-19 was identified as the leading cause of death in about half of these cases, but also heart disease, neoplasms, cerebrovascular diseases and Alzheimer disease and other dementias were commonly associated with these deaths. Older persons and men seemed at higher risk for Post-COVID related death. Post-COVID mortality is higher in the northern area of Italy and decreases in the South, following the distribution of the COVID-19 cases and deaths in the Country (19).

The overall number of deaths and mortality rate in the Italian population seems substantially higher as compared with what described in a recent study performed in the US (13). These differences can be due to the different methodology adopted in the two studies. As mentioned, in the study performed in the US, the identification of Post-COVID deaths was based on literal text search of death certificates, since the ICD-10 code to identify this condition was not implemented in the period considered in the analysis, leading potentially to an underestimation of Post-COVID deaths. At the opposite the Italian National Institute of Statistics (ISTAT) adopted this code and made it available for use in death certificates from September 2020, allowing for a more careful coding of Post-COVID condition.

Post-COVID condition was poorly known at the beginning of the epidemic and the first study recognizing and reporting this condition in the Italian population was published in July 2020 (21). First guidelines on this condition were published between the end of 2020 and beginning of 2021 (17, 22). In addition, poor availability of diagnostic tools for COVID-19 in the first 2 months of the epidemic in Italy (March and April 2020) may have limited the ability to diagnose not only COVID-19 but also its long-term consequences in 2020 (23). Therefore, a misclassification of Post-COVID related deaths, particularly in 2020 is possible and for this reason only mortality rates for year 2021 are presented in this study.

Long term consequences of COVID-19 can substantially impact on health outcomes. In a cohort study performed on more than 600,000 adults in Italy, patients suffering from COVID-19 had a 2-fold higher rates of outpatient visits and hospitalizations and nearly 3-fold higher rates of instrumental diagnostic procedures in the 6-month after acute the infection (9). Similarly, in a cohort study performed in the US on more than 100,000 patients, SARS-CoV-2 infection was associated with a 4% increase in healthcare utilization over a 6-month period, mainly for emergency department visits (24). COVID-19 has been associated with onset of acute conditions that can increase mortality in the long term (i.e., stroke or myocardial infarction) or it can lead to progressive onset of severe and potentially life-threatening in the long-term, such as myocarditis or dementia (7, 8).

Noticeably, in almost half of the cases of Post-COVID related deaths the underling cause of death was represented by a chronic condition, including heart disease, neoplasms, cerebrovascular disease and Alzheimer dementia. This finding underlines the fact that COVID-19 can interact with pre-existing conditions, leading to increase long-term mortality due to these conditions (25). The interplay between COVID-19 and pre-existing health conditions can lead to increased mortality rates by exacerbating underlying health issues, compromising organ function, and impairing the body’s ability to fight off the infection.

Interestingly men and older adults have the highest mortality rate for post-COVID condition. This finding mirrors what was observed for acute COVID-19 mortality, suggesting a common pathway leading to increased mortality related to acute and long-term consequences of SARS-CoV-2 infections, in persons with these characteristics (2, 3, 26). Advanced age is associated with the presence of multiple chronic conditions and with frailty, which can increase the susceptibility to negative health consequences related to COVID-19 and Post-COVID (3). Several factors can explain gender differences in COVID-19, which can be generalized to post-COVID (27, 28). Biological factors can make men more susceptible to severe outcomes from certain infections, including COVID-19, while women generally present a stronger immune response. Men have a higher prevalence of underlying health conditions, including cardiovascular disease, hypertension, and diabetes, which increase the risk of severe COVID-19 outcomes. Finally, hormonal differences could play a role in the immune response to infections.

The present study has several limitations. First, the ICD-10 code identifying post-COVID conditions was implemented at a national level on September 2020 and therefore deaths associated with this condition occurring before this date may have been underestimated. Second, we can not link Post-COVID related deaths with SARS-Cov-2 infection and therefore it is not possible to measure the time interval between SARS-CoV-2 infection and Post-COVID related deaths and to assess how infection characteristics (i.e., infection severity or variant) impacts on mortality. Indeed, the onset and time course of conditions largely varies across individuals and by type of condition (7, 8, 29). Neurological conditions often have a delayed onset of weeks to months and several neurocognitive symptoms can worsen over time. Similarly, cardiovascular and respiratory complications of COVID-19 infection can progressively worsen over time, leading to increased mortality in the long-term. Third, data presented apply to the Italian population and cannot be generalizable to other countries or regions with different healthcare systems, demographic profiles or varying degrees of SARS-COV-2 infection spread. In this context, we described substantial differences in Post-COVID-related mortality between Italy and the US. Further analyses from death registries of additional countries may be necessary to comprehensively address this topic. Finally, while death certificates can provide valuable information for epidemiological research, they may have not captured the full complexity or nuances of post-COVID conditions. This could be attributed to the limited knowledge on the numerous potential long-term consequences of COVID-19, particularly during the early phases of the epidemic, and the possibility that some conditions leading to long-term mortality from COVID-19 may not have been accurately diagnosed as related to the infection.

## Conclusion

In conclusion, we show that Post-COVID condition contributed to a substantial number of deaths in Italy. Strategies to identify the population at risk of severe long-term consequences of SARS-CoV-2 infection and interventions aimed at assessing this population and reducing this risk must be developed.

## Data availability statement

The analyses presented in the paper are based on aggregated data. Requests for access to the causes of death dataset should be addressed to the Istat contact centre (https://contact.istat.it/s/?language=en). The datasets presented in this article are not readily available because the data analysis used in this study complies with the European General Data Protection Regulation (GDPR EU 2016/679). The Italian Data Protection Authority has authorised the processing of personal data on causes of death by the Italian Institute of Statistics.

## Author contributions

FG: Data curation, Methodology, Software, Supervision, Writing – original draft, Writing – review & editing. GM: Methodology, Supervision, Writing – review & editing, Writing – original draft. RC: Supervision, Writing – review & editing. SM: Methodology, Supervision, Writing – review & editing. FP: Supervision, Writing – review & editing. GO: Supervision, Writing – original draft, Writing – review & editing.

## Footnotes

^1^ www.iris-institute.org

## Appendix

**Table A1:** Emergency ICD-10 codes for COVID-19 and post-COVID.

Codes	Description	Positions in death certificates
U07.1	Confirmed diagnosis of COVID-19	Underlying cause
U07.2	Clinical or epidemiological diagnosis (suspected or probable) of COVID-19	Underlying cause
U10.9	Multisystem inflammatory syndrome associated with COVID-19, unspecified	Other causes
U09.9	Post COVID-19 condition	Other causes

The underlying cause of death is defined as ‘the disease or injury which initiated the train of morbid events leading directly to death, or the circumstances of the accident or violence which produced the fatal injury’. Codes U10.9 and U09.9 cannot be used for the underlying causes of death and if reported as underlying causes of death, according to WHO provision, the death was attributed to COVID-19.

**Table A2:** Deaths and standardized mortality rates (deaths per 100 thousand inhabitants) of Post-COVID condition and all causes by sex and area (Nuts1) of residence (Year 2020 and 2021).

Nuts1 Area	Deaths						Standardized mortality rates		
	2020			2021			2021		
	Men	Women	Total	Men	Women	Total	Men	Women	Total
Post-COVID-19 condition									
North-west	112	160	272	540	659	1,199	6.3	4.5	5.3
North-east	67	106	173	496	610	1,106	7.9	5.7	6.8
Center	57	65	122	371	420	791	5.8	3.9	4.8
South	52	45	97	340	362	702	5.1	3.8	4.4
Islands	19	20	39	123	113	236	3.8	2.3	3.0
Nonresidents, missing	2	1	3	6	6	12			
Italia	309	397	706	1,876	2,170	4,046	6.0	4.3	5.1
All causes of death									
North-west	54,541	57,620	112,161	90,640	97,177	187,817	1,082.1	696.1	854.9
North-east	37,062	40,719	77,781	65,084	70,194	135,278	1,060.2	689.1	844.7
Centre	38,012	40,758	78,770	69,369	74,951	144,320	1,089.4	722.8	877.1
South	41,418	41,275	82,693	77,937	80,503	158,440	1,198.8	820.2	984.9
Islands	20,806	20,651	41,457	38,579	39,898	78,477	1,201.8	818.4	983.5
Nonresidents, missing	961	570	1,531	1,734	903	2,637			
Italia	192,800	201,593	394,393	343,343	363,626	706,969	1,117.2	738.8	898.5
